# Medical Miscellanies

**Published:** 1816-06

**Authors:** 


### PART IV.

### MEDICAL MISCELLANIES.

*Medical Biography.*—We have formed, from materials furnished us by *Leroux's Journal* and the *Gazette de Santé*, the following slight biographical sketches of two distinguished medical philosophers, lately deceased in France. We are accumulating matter for similar notices of eminent professional men, both British and continental; and shall gladly receive from Correspondents any contributions to our stock. This species of composition possesses a charm, and exerts an influence on the human mind, which signally entitle it to cultivation in a Philosophical Journal. It affords a pleasing recreation to the intellect, exhausted by rigorous research into more abstruse branches of study; and nothing can more effectually reconcile the young " spirit struggling for distinction" to the ruggedness of the path, nothing more powerfully rekindle the dormant spark of enthusiasm, or sustain the staggering resolution, amid the ' chills and bruises" which must necessarily be encountered, than the spectacle of those illustrious men who, triumphant over every difficulty, have reached

" The hill where Fame's proud temple shines afar;"

and at last descended to the grave, universally honoured and lamented.

1. *Jacques Tenon*, the patriarch of the present race of French Surgeons, was born in 1724. His father practised surgery. His mother was a sensible and accomplished woman; and to her the charge of young Tenon's early education was committed. In 1741, he repaired to Paris. Here he soon attracted the notice of Winslow, and of Antoine and Bernard de Jussieu. By these celebrated men he was instructed in the sciences of anatomy and botany.

French surgery had just been re-established by Louis XV. in all its honours and literary prerogatives. Tenon, in order to facilitate his progress, devoted himself with ardour to the cultivation of the Latin language, and acquired the necessary title of Master of Arts. He was appointed, in 1744, assistant surgeon-major to the army in Flanders, and discharged the duties of his situation with zeal and fidelity. On his return to Paris, he obtained the situation of chief surgeon to *L'Hôpital de la Salpêtrière;* not far from whence he opened a house for inoculation, which soon became celebrated. Tenon, in fact, was one of those who contributed most to establish this practice, then novel, in France; and yet he hesitated not to relinquish it for vaccination, when convinced of the superiority of the new process. He, soon afterwards, became member of the College, and Royal Academy, of Surgery; having first written, and publicly defended, a Latin thesis upon cataract, remarkable for soundness of doctrine and purity of style. He succeeded in a short time to the chair of pathology, vacated by Andouillé.

In a few years, Tenon acquired great celebrity. Students, destined for the service of their courts and armies, were frequently sent to him by foreign sovereigns: at the age of 33, he was received into the vacant place of the celebrated J. L. Petit, in the Royal Academy of Sciences. Here he gained a complete, though long-disputed victory, over a party who wished to suppress the anatomical class. His attention was next turned to the Parisian hospitals; the construction of which it was his object to render more healthy and commodious, and to establish in their administration greater order and œconomy. *L'Hotel-Dieu* he wished to remove from the centre of the city; but failing in the attempt, he attacked, with energy and success, the abuses which prevailed in that immense edifice. Called to the first legislative assembly, he there displayed, in the cause of humanity, all the enthusiasm and philanthropy of his character. No one ever more clearly traced the causes, effects, and remedies of beggary: the orphan, the foundling, the aged, and infirm, found in Tenon a zealous advocate. Solicitude for science and the public good engrossed all the powers of his frame and intellect. His house could scarcely contain all the drawings, the ancient and modern instruments, anatomical preparations, specimens of extraordinary and diseased bone, and models of all kinds of apparatus, which he was daily accumulating.

Tenon was frequently in the habit of retiring to a country residence near Paris: but this repose, which had been frequently broken in upon during the revolution, was, at length, utterly destroyed. His farm at Massy was occupied by foreign troops, and, during this period of ruin and disorder, he had the mortification to witness the destruction of his favourite library. This misfortune affected him deeply, and. doubtless, hastened his dissolution. In January, he went, as well as usual, to a public session of the Institute, but caught cold in returning: on the day after, he was affected with fever and oppression; and calmly expressed to Percy and Huzard, who visited him in the name of the Society of the Faculty of Medicine, the consciousness of his approaching end. At four o'clock in the morning of Monday, January 15th, the venerable and philanthropic Tenon died, aged 92 years. He was never married. The principal works published by him are, a *Memoir upon the exfoliation of bones; Memoirs upon the hospitals of Paris;* and one *upon the developement of the teeth.*

2. *Jean-Jacques Menuret de Chambaud,* Doctor in Medicine, died about the close of 1815, aged 82 years. Born in 1733, at Montelimart, he commenced his medical studies, when very young, at the school of Montpellier. He was afterwards initiated into practice under Dr. Fise, at that period a physician of considerable eminence in the province of Languedoc. The preceptor of young Menuret was a learned but eccentric man, and seems to have possessed an unusual share of sagacity and penetration. He judged of a patient's disease by his pulse, and hesitated not to tell him that he would recover, or to pronounce his doom in a very unreserved and often abrupt manner; and he is *said* to have been rarely mistaken. In 1768, Menuret published his *Traité sur le Pouls* (Treatise on the Pulse); and, soon afterwards, his *Avis aux Meres sur la Petite-verole et la Rougeole* (Advice to Mothers respecting Small-pox and Measles). His other medical works are, *Essai sur l'Action de l'Air dans les Maladies contagieuses* (Essay on the Influence of the Air in contagious Diseases); *Essai sur l'Art*

*de former les bons Medecins* (Essay on the Art of forming good Physicians); *Essai sur la Ville de Hambourg*,* &c.; and *Histoire Medico-topographique de Paris*. He was also author of a memoir on the topography of Montelimart, inserted in Richard's *Recueil des Observations sur les Hôpitaux;* and of several good articles in the Encyclopœdia. He was a man of the most active benevolence; and his exertions in the cause of humanity ceased but with his life.

*Hernia.*—1. *Case of crural hernia, in which were contained the uterus, ovaries, and part of the vagina.* On the 15th of December last, a woman, aged 82, who, for forty-two years, had carried a large hernial tumor of the right side, was admitted into *La Salpêtrière* with all the symptoms of strangulation. She was mother of eight children. For several years, she had been very subject to nausea, colics, and a sense of dragging of the stomach. The tumor, considered *enterocele*, had been reduced, and confined by a bandage, which, for some years, was negligently worn, and at last abandoned The tumor, thus left to itself, increased in volume, and became irreducible. The woman sometimes vomited after food.

"Such was the habitual state of the patient till her 74th year. At that period she was affected with acute fever. During a paroxysm of delirium, the tumor acquired suddenly a double size, and some symptoms of strangulation were developed; as colic, hiccough, nausea, and even vomiting. The abdomen was tense; the tumor hard, and the integuments displayed some traces of inflammation. Poultices were applied to the tumor: after a few days, fluctuation was perceptible; and the surgeon, who believed the hernia to be *enterocele*, and that its size forbade an operation, contented himself with combating the inflammation by cooling ptisans. The sensible presence of fluid towards the right of the tumor induced a suspicion of stercoral abscess. At length, the inflamed integuments gave way; and there escaped, not pus or fæcal matter, but a serous fluid somewhat bloody and extremely copious. This discharge was followed by diminution in the volume of the tumor. The symptoms of strangulation disappeared, as well as the antecedent fever."

---

* Or *Letters on the Medico-topographical History of that City*. This work, written in 1797, was the fruit of Menuret's exile to Hamburgh; whither he had been compelled to take refuge from the following circumstance: He was physician to Dumourier, when that general received an order from the Convention to repair to Paris, in order that his conduct might be investigated. This order was no less than a sentence to the scaffold. Dumourier, embarrassed and irresolute, said to Menuret, as he entered the room, "Well, Doctor, have you any remedy for this complaint?"—"Oh yes, General, a little prescription of *disobedience*." Dumourier took his physician's advice, but imprudently published the anecdote; and the poor doctor was obliged to run for his life.

From this time to the present, the patient remained subject to nausea, pains of the belly, sometimes vomiting: and, about once every three months, the tumor increased in size, the integuments burst, and gave issue to an inodorous fluid of an oily consistence; the expulsion of which produced a diminution in the volume of the hernia.

On the 20th of December, the day after her admission into *La Salpétrière,* she exhibited fever, nausea, slight vomiting, colic, constipation. " The tumor offered the following peculiarities: it was seated in the right groin; its volume was very great, about five inches in length, and four in breadth. Its form was that of a pyramid with three faces: one of these was anterior; another, posterior, rested upon the right thigh; the third, internal, passed some inches beyond the *vulva.* The base was above; the summit below; and the tumor was broader in the middle than at the base: its direction was oblique from right to left, and from above downwards. The integuments had yielded to such a degree, that the tumor formed a sac suspended between the thighs. The finger, passed above it, distinguished the abdominal ring in a natural state; and immediately below was felt the crural arch."

The size of the tumor and slightness of the symptoms of strangulation, with the age of the patient, were thought to contraindicate the operation. Mild laxatives and emollient injections were therefore only employed, and they served to allay the symptoms: there was now also a discharge of serum from the integuments of the hernia, without any fistulous opening, and too copious to be mistaken for perspiration. Scarcely had the symptoms of strangulation subsided, when the woman was attacked with asthenic fever, which shortly proved fatal. The following appearances were exhibited on dissection:

" The skin which formed the external covering of the tumor was sound. Below existed a considerable mass of fat, of firm and close consistence. The hernial sac could scarcely be distinguished. From the long existence of the hernia, the natural state of the parts was lost. More deeply, we discovered a lardy adipose texture in considerable quantity, to which were attached two elongations of omentum. This part was divided, and formed into two flaps, internal and external: no intestine was then seen, but the following objects, adherent on all sides to the surrounding fat: The uterus, ovaries, Fallopian tubes, part of the vagina, two distinct bands of omentum, and two cysts, or perhaps hydatids. In order to examine correctly these parts, we exposed the abdominal ring, cut the ligament of Fallopius, and clearly distinguished a crural hernia. The epigastric artery was exterior to all these parts, which themselves lay anteriorly to the united tendons of the *iliacus internus* and *psoas* muscles: they rested, in their passage, on the most internal part of the horizontal ramus of the pubis; the crural vessels were consequently external and posterior to the displaced parts.

" These parts were thus disposed: in examining from left to right, we saw, 1st, a band of omentum stretching from the transverse colon, and losing itself below in the mass of fat at the inferior part of the tumor; 2dly, a second band of omentum, somewhat more to the right, terminating below as the former, and coming from a point of the transverse colon, nearer its ascending portion;—these two bands, the last of which had a fleshy appearance, seemed to draw down the arch of the colon: 3dly, still farther to the right and above, the Fallopian tube; inferiorly, the right ovary and broad ligament, which was contracted: 4thly, the uterus, its cervix above, perceptible through the parietes of the vagina, when pressed with the finger; its fundus, turned downward, corresponded to the fatty mass before described;—the finger, introduced into the *vulva*, detected an unnatural direction of the vagina from left to right; a probe, passed by this opening, penetrated beyond the crural arch, so that the vagina, much elongated, formed the hernia by its superior part: 5thly, the right Fallopian tube, part of the broad ligament, and the right ovary disorganized and presenting a kind of cyst; below, a second cyst, independent of the former, or perhaps an hydatid, to which certainly might be attributed the flow of serum during the patient's life: lastly, quite to the right was found an abundant cellular structure. The bladder and rectum were in their natural situation; their functions had never been deranged." *Professor Lallement. Bulletin de la Faculté de Médecine de Paris, &c.* 1815, No. x.

2. *Artificial anus spontaneously cured.* A robust woman, aged 48, had been, for several days, affected with all the symptoms of strangulated inguinal hernia. The operation, proposed by the surgeon, was resolutely rejected by the patient and her friends. When he left her in the evening, the integuments of the tumor had acquired a blackish hue; the pulse was very feeble; the body covered with cold sweats: low delirium had come on.—" On the following morning, my surprise was great, when informed that the woman was better, and had recovered her reason; and that the inclination to vomit had immediately ceased upon the discharge of two large worms, and fæcal matter from the tumor. I ascertained the fact, and found the intestine perforated, and a wound of gangrenous character, about the size of a crown of three Francs. I dressed with the *baume de Geneviève* (a composition of wax, turpentine, oil, red wine, and camphor), and preserved the freedom of the belly by injections. A tonic regimen was also prescribed, and some anthelmintic remedies fruitlessly administered, as no more worms were voided. This treatment, pursued three weeks, gave energy to the system: the margin of the wound became red, and covered with granulations; the circumference of the slough formed adhesions to the surrounding parts; the wound contracted; and at the end of two months was completely cicatrized. The fæces have always been readily voided by the natural channel. The woman has now remained quite well, during four years." *Dr. Dabry, Lyons. Gazette de Santé, February* 1816.

*Case of Excision of two of the Metacarpal and Carpal Bones, followed by the Formation of Anastomosing Aneurism (communicated to Dr. Palmer).* A young woman, of decidedly strumous family and habit, had long been affected with thickening and ulceration of the left hand, on its radial margin, and evidently connected with a morbid state of the metacarpal bones of the thumb and fore-finger. As all remedies, constitutional and topical, had been tried in vain; as the disease was progressively extending towards the carpus, and the system of the girl beginning to be re-acted upon by the irritation of the local malady, an operation was, at length, proposed and acceded to. Her situation in life rendered it an object of importance to save, if possible, part of the member. It was, therefore, resolved, first to remove the thumb and fore-finger at their articulation with the carpus, and only to sacrifice the hand, if the disease should be found to have extended farther than was supposed. The integuments of this part of the hand were so much diseased that little could be saved. The metacarpal bone of the middle finger was sound; but the trapezium and trapezoides were found black, and clearly in a diseased state. This was embarrassing, for excision of two of the carpal bones was a measure of which we knew not a precedent; and the doctrine of the schools led us to anticipate dreadful mischief from introduction of air into the interior of the carpal capsule. But circumstances warranted an experiment; and that experiment was determined on. The morbid trapezium and trapezoides were removed. Great embarrassment was encountered in securing the vessels: no sooner was one small vessel tied, than another sprang; so that the application of thirteen or fourteen ligatures was necessary. From the loss of integuments, the wound was nearly reduced to the state of an open sore. In the course of the evening after the operation, it became hot and painful; and continued so all the next day. Cooling lotions were prescribed. On the third day, profuse hæmorrhage came on; but again subsided on exposing the wound. In this way, we were harassed, day and night, for more than a week. All the common precautions, and means of arresting hæmorrhage, were employed in vain. At length, the young woman's strength was so much impaired, and her mind so strongly impressed with the dread of bleeding to death, that some decisive measure was imperiously demanded: and it was resolved, in the event of a recurrence of the hæmorrhage, to pass a ligature first round the trunk of the radial artery, and afterwards to tie, if it should be necessary, the ulnar. But, fortunately, the operation was not required; for, about this time, the bleeding began greatly to decrease in frequency and violence. It had not returned for about five days when I, one morning, called to inspect the progress of the wound. It had contracted very little, and had a languid unfavourable appearance; but what particularly arrested my attention, was a *purple, spherical tumor*, about the size of a hazel nut, seated on the surface of the sore, near the head of the third metacarpal bone, *and pulsating violently.* Upon inquiring of the young gentleman, who had dressed the wound, I found that this tumor had first appeared, four days before, in the form of a "flabby granulation" (which he, in fact, supposed it to have been); that it must have increased very rapidly during the last twenty-four hours; and that he had never before observed it to display a pulsatory action. While I stood examining it, the tumor burst suddenly at the centre, and a stream of *florid arterial* blood sprang, in a *regular uninterrupted stream*, from the orifice. I placed my finger upon it, and the tumor again pulsated strongly. A ligature was now passed round the base; but, on tightening it, the little tumor separated, and the bleeding re-commenced from the parts beneath with violence. Every effort to arrest it was useless, until a strong ligature had been passed through the surrounding soft parts, and firmly tied on a small roll of lint, applied to the seat of the hæmorrhage. The separated part was formed of a dense, purplish structure, *and seemed to consist of an infinite number of very minute cells.* From that period, the bleeding never returned; and the wound got slowly, but progressively, well. When I last saw the young woman, her health was restored; and she found her mutilated hand extremely useful. The case appears to me to be, in many respects, valuable.

*Case of sudden death, with some unusual symptoms. By one of the Editors.*—A woman, of rather corpulent figure, about 50 years of age, and who had a few months before, suffered a paralytic affection of the lower extremities, from which she had recovered, was seized, at five o'clock in the afternoon, with a sudden and excruciating pain in the left arm, extending from the axilla to the elbow, in the line of the nerves and humeral artery. About an hour afterwards, one of the Editors visited her. The whole arm and fore arm were now insensible, excepting along the line of the great vessels, where pressure still gave some pain. There was no pulsation at the wrist, nor, as far as could be ascertained, in the humeral artery. The temperature was much fallen; the fingers pale and contracted; and no power whatever in the muscles: so that the arm lay dead along side of her. Still she experienced such intense pain in the part already mentioned, as to cause her to cry out in great agony. The pulse at the other wrist, was 150, but irregular. The heart beat violently and tumultuously against the ribs—the carotids and temporals were in unison with the radial artery of the right arm, but the whole vascular system appeared in a state of the utmost tumult. The nervous system was generally unaffected; the eye perfectly natural—the pupils dilating and contracting freely—the senses entire and undisturbed, except from the pain in the arm. Abdominal pressure gave little uneasiness, except at the epigastrium, where some tenderness was felt; but there was no tension—no heat of the surface, nor any symptom of pyrexia, save the quick and highly irregular pulse. The bowels were torpid; the tongue moist; no thirst; all the muscles of voluntary motion under command, excepting those of the left upper extremity. The state of the respiration corresponded with that of the circulation; it was hurried, short, and somewhat laborious. The face was neither flushed nor pallid; yet indicated much anxiety. Cathartics operated scantily; frictions with stimulating liniments, produced no effect on the arm. Æther and opium at bed time, gave neither ease nor sleep.

To the foregoing symptoms were, next day, added, nausea and vomiting. Her state in other respects, was precisely as is above described, excepting an *intermitting* pulse. A consultation was now held, but the nature of the disease could not be ascertained. It was determined, however, to try the effect of venæsection, as the case was desperate, and death seemed approaching. The blood sprang freely from the vein, and while it was flowing, several medical men kept their fingers on the carotids, temporals, &c. to ascertain the effects of this *anceps remedium.* Fifteen or sixteen ounces were in the bason, but not the slightest alteration was observeable in the state of the vascular system — gastric irritability — or dolor brachii. It was judged unsafe to proceed further. Five grains of the submuriate of quicksilver were given, and ordered to be repeated in six hours; and sinapisms were applied to the soles of the feet.

At ten o'clock next morning, she was insensible, and at one P. M. or forty-four hours from the commencement of the attack, she expired.

No entreaties could prevail on the family to permit an inspection of the body; but immediately after death, the integuments about the *left clavicle* turned quite black.

No suspicion of aneurism existed during her life; but that some sudden rupture of an artery or aneurism in the subclavian region occasioned the train of symptoms enumerated, was the opinion of the most intelligent of the medical attendants. The *sensorial* system was quite unaffected till near the close of life, when it, of course, participated in the fatal derangement of the vascular system. The blood drawn exhibited no inflammatory appearance, though abstracted in a manner calculated to show the marks of inflammation, if any such existed. This remark is made because it was hinted by one or two medical gentlemen, that the disease was merely inflammation of the *stomach.* The absurdity of such a suppposition is unworthy of refutation; but it shews into what preposterous errors even *experienced* (at least *soi-disant* experienced) practitioners will occasionally fall, when they suffer *self-conceit* to usurp the place of legitimate study — accurate observation, and cautious investigation!

The Editors solicit the candid opinion of their friends and correspondents on the above case

The Medical Benevolent Society.—The Directors of this truly liberal and philanthrophic Institution, which embraces the interests of every medical practitioner in the United Kingdom who is a member of it; having, with the best legal assistance, finally arranged the laws and regulations by which it is to be governed; the Society is now permanently established. Many liberal donations and numerous subscriptions have been received, forming in the aggregate a considerable sum of money, conformably with the purposes expressed in the Prospectus. Further particulars of the Society may be known, by applying (post paid) to Mr. BEST the Secretary, 15, Tavistock Street, Covent Garden.

On Thursday the 30th of May, Dr. J. B. Davis was elected Physician; Mr. Pettigrew and Mr. Gilham, Surgeons; and Mr. Wasdell extra Surgeon to the Universal Dispensary for Children. This Institution, established for the exclusive treatment of infantile diseases, is indebted for its origin to Dr. Davis, who by his unwearied exertions in its formation and progress, has at length succeeded in rendering it efficient for the public service.

The Museum of the Royal College of Surgeons will be open for inspection on Tuesday the 11th day of June next, and on every Tuesday and Thursday from such date, until the last Thursday in August. For particulars relating to admission, application may be made at the College, between the hours of twelve and three.

Mr. T. J. ARMIGER, Surgeon, &c. has just published a new Work, entitled "Rudiments of the Anatomy and Physiology of the Human Body; consisting of Tables, &c. compiled for Students of those sciences beginning their researches.

Mr. HOWSHIP has in the press, nearly ready for publication, some "Practical Observations on the Diseases of the Urinary Organs," illustrated by Cases and Engravings.

Mr. JOHN TAUNTON's Summer Course of Lectures on Anatomy, Physiology, Pathology, and Surgery, commenced on Saturday, May 18th, 1816, at eight o'clock in the evening, precisely, and will be continued every Tuesday, Thursday, and Saturday, at the same hour. Particulars may be had on applying to Mr. Taunton, 87, Hatton Garden.

Mr. CARPUE will commence his Course of Lectures on Anatomy and Surgery on Monday the 10th of June.

DIED.—Aged 56, Dr. John O'Donnell, of Welbeck Street, Cavendish Square, late of Uxbridge.

